# A Field Trip to the Archaean in Search of Darwin’s Warm Little Pond

**DOI:** 10.3390/life6020021

**Published:** 2016-05-25

**Authors:** Bruce Damer

**Affiliations:** 1 Department of Biomolecular Engineering, Baskin School of Engineering, University of California at Santa Cruz, Santa Cruz, CA 95064, USA; bdamer@ucsc.edu; Tel.: +1-831-459-2158; 2 DigitalSpace Research, Boulder Creek, CA 95006, USA

**Keywords:** origin of life, hydrothermal systems, progenote, microbial communities, stromatolites

## Abstract

Charles Darwin’s original intuition that life began in a “warm little pond” has for the last three decades been eclipsed by a focus on marine hydrothermal vents as a venue for abiogenesis. However, thermodynamic barriers to polymerization of key molecular building blocks and the difficulty of forming stable membranous compartments in seawater suggest that Darwin’s original insight should be reconsidered. I will introduce the *terrestrial origin of life hypothesis*, which combines field observations and laboratory results to provide a novel and testable model in which life begins as protocells assembling in inland fresh water hydrothermal fields. Hydrothermal fields are associated with volcanic landmasses resembling Hawaii and Iceland today and could plausibly have existed on similar land masses rising out of Earth’s first oceans. I will report on a field trip to the living and ancient stromatolite fossil localities of Western Australia, which provided key insights into how life may have emerged in Archaean, fluctuating fresh water hydrothermal pools, geological evidence for which has recently been discovered. Laboratory experimentation and fieldwork are providing mounting evidence that such sites have properties that are conducive to polymerization reactions and generation of membrane-bounded protocells. I will build on the previously developed *coupled phases* scenario, unifying the chemical and geological frameworks and proposing that a hydrogel of stable, communally supported protocells will emerge as a candidate *Woese progenote*, the distant common ancestor of microbial communities so abundant in the earliest fossil record.

## 1. Introduction

Most workers investigating the origin of life confine their horizons to theoretical papers, computational models or laboratory simulations that use a narrow spectrum of commercial reagents reacting in buffered aqueous solutions. The results are then related to prebiotic conditions with the assumption that laboratory or *in silico* results can plausibly occur in the early Earth environment. A complementary approach is to visit sites that are analogs to candidate venues for biogenesis. Such fieldwork broadens our perspective by providing a more realistic assessment of the actual conditions in which physical and chemical reactions progressed toward life’s beginning approximately four billion years ago. Furthermore, geologists familiar with the most ancient, Archaean fossil sites can interpret the rock record and provide knowledgeable advice about geochemical conditions to guide model building and laboratory experiments.

## 2. A Field Trip in Deep Time to the Archaean

**Figure 1:**
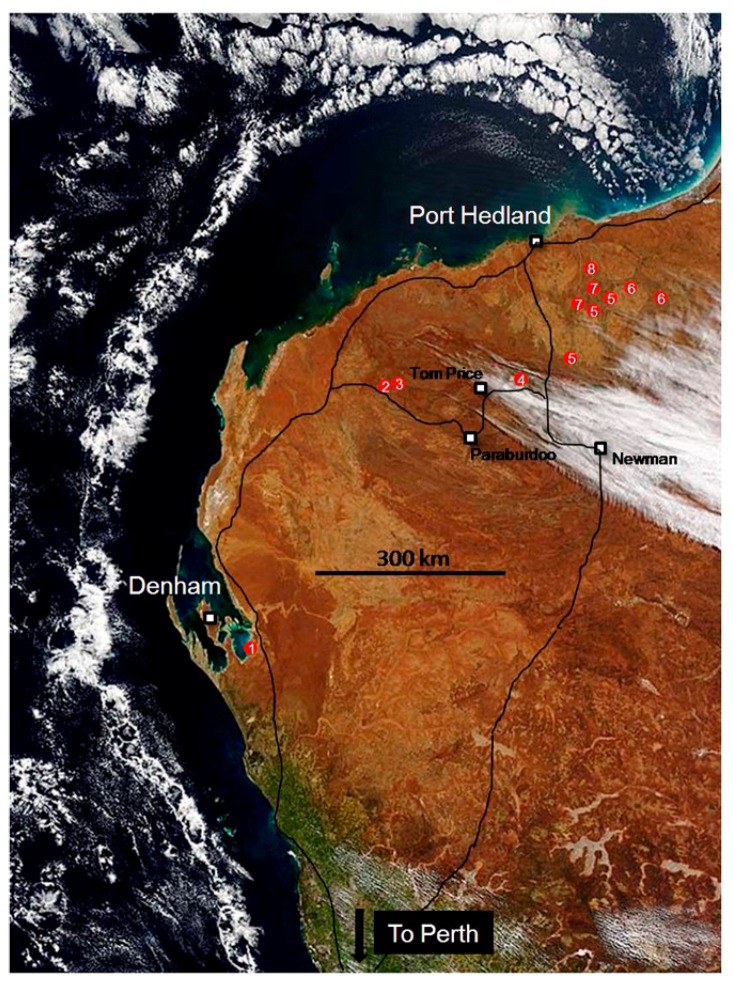
Route taken from Perth, Western Australia to Shark Bay near Denham, north via Paraburdoo and Tom Price into the Pilbara region before departure from Port Hedland. Sites visited are marked in red (1–8). Source: Government of Western Australia Department of Mines and Petroleum.

In July 2015, I joined a unique scientific field trip led by Malcolm Walter and Martin Van Kranendonk of the Australian Centre for Astrobiology at the University of New South Wales. This 3000 km trek (Figure 1) by air, bus, utility vehicle and foot traveled through 3.5 Ga (billion) years of Earth’s history across Western Australia, from Perth to Hamelin Pool (1) at Shark Bay, northward through the Hamersley Range and Fortescue Group (2–4) to the Marble Bar area (5) and finally to sites (6–8) in the remote North Pole Dome of the Pilbara Craton. During the ten-day tour, new ideas emerged through examination of evidence from the earliest known, Archaean geological settings inhabited by microbial life.

## 3. Living Marine Stromatolites and Inland Microbial Communities

**Figure 2:**
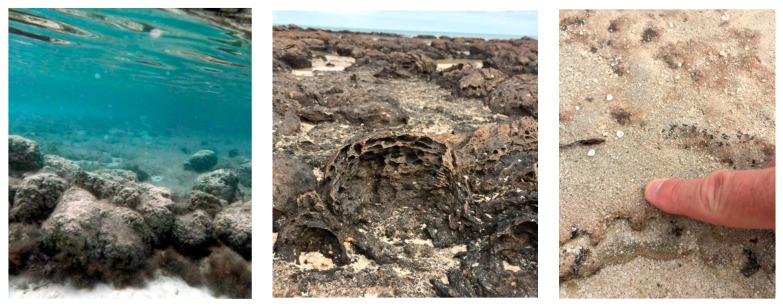
(**Left**) Underwater view of stromatolites in Hamelin Pool, Shark Bay, Western Australia (credit: Marisol Juarez Rivera). (**Center**) Eroded section through a recently exposed Shark Bay stromatolite, showing the domical shape and crude laminations of these large, lithified structures (width of central stromatolites is ~20 cm). (**Right**) Oblique view down onto silt and sand-covered, living microbial mat from Shark Bay. The ragged textured edge in the photo is an eroded section cut through the microbial mat, which exhibits rubbery cohesion as it is composed of a community of microorganisms. These contemporary versions of the Earth’s most ancient microbial ecosystem were later compared to Archaean stromatolite fossils. Center and right photos by author.

The journey began with a visit to one of the best examples of living stromatolites, in Hamelin Pool at Shark Bay (Figure 2). These shallow water domical and mat-like structures are formed by microbial communities under conditions of elevated salinity that prevents larger organisms from feeding on the communities. The organisms that form stromatolites cause carbonate minerals to precipitate in distinctive layers, forming structures that can be preserved for billions of years in the fossil record.

**Figure 3:**
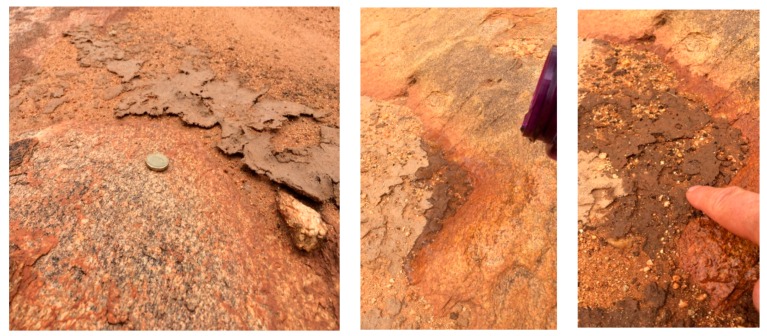
(**Left**) Author photos of microbial mats growing on exposed rocks at Gallery Hill; and (**Center**) and (**Right**) hydrating a mat and testing its plasticity.

The stromatolites produced by microbial communities at Shark Bay require near-continuous immersion in a marine environment. By way of contrast, terrestrial microbial mats were also observed on the exposed rock surfaces of Gallery Hill in the semi-desert Pilbara region near Marble Bar (Figure 3). Hydrating a dry mat (by simulated rainfall) showed that the mat quickly absorbed the water and retained it far longer than surrounding mineral surfaces (as documented elsewhere by Verrecchia *et al.* [1]). Significantly, the moistened mat had the same physical consistency as the living stromatolite mats at Hamelin Pool. This microbial community inhabited a rock-soil margin resembling a shoreline and depended on infrequent precipitation.

Could there have been an evolutionary link between marine stromatolites and these primitive microbial mats pointing back to a common ancestor depending on a more reliable fluctuating source of fresh water? If so, how could these fresh water mats have evolved to survive extensive dehydration and exposure to the much higher ultraviolet (UV) component of Archaean sunlight?

**Figure 4:**
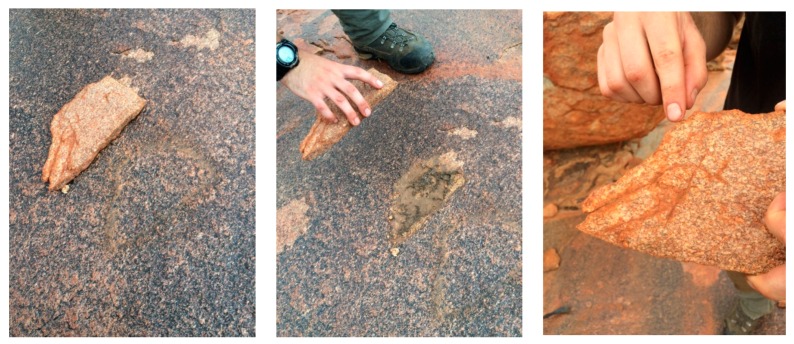
Author photos of rock next to an impression of its earlier resting place on Gallery Hill (**Left**); microbial community in moist sediments beneath it (**Center**); and view of rock underside (**Right**).

A possible answer was suggested by examination of one of the many flat rocks littering the surface of Gallery Hill (Figure 4). The rock lay on a weathered surface covered by what is known as “desert varnish”, a favorite canvas for Aboriginal artists. Beneath the rock was a microbial community thriving in moist sediments (center), consisting of cyanobacteria shaded from the ultraviolet component of sunlight but able to gather enough diffuse light for metabolism (as noted elsewhere in the Pilbara by Hoshino and George [2]). On the early Earth, shade provided by rocks and minerals would have protected against ultraviolet light and retained moisture essential for life. Cracks within mineral surfaces (right) can also provide such protection for cryptoendolithic microorganisms that thrive today in the Antarctic high deserts [3].

**Figure 5:**
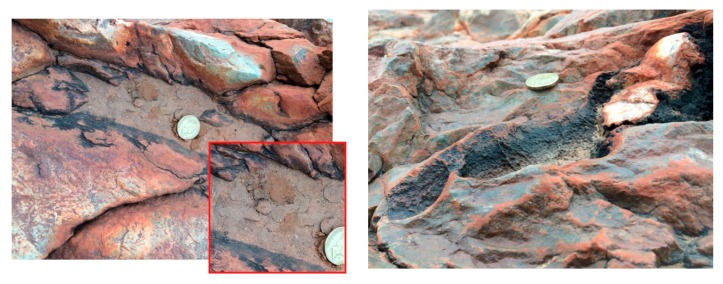
Author photos of rock-crevice pool (**Left**) at Marble Bar concentrating moisture and organic solutes capable of supporting microbial mats (**Inset**). (**Right**) Dried pool illustrating microbial chemical staining on the rock surface.

These observations suggested an interesting comparison between two sites proposed for the origin of life: submarine hydrothermal vents [4,5,6,7,8] and hydrothermal fields on exposed land surfaces [9,10]. Assuming a source of organic compounds such as amphiphiles, amino acids, and nucleobases supplied by extraterrestrial infall [11,12,13] or geochemical synthesis [14,15], these compounds would either be present as a dilute solution in the Archaean ocean [16] or deposited on volcanic land masses. Exposed rocky surfaces that support localized pooling from precipitation would provide a venue for early microbial communities, as illustrated by the isolated pools on the *ca.* 3.4 Ga jasper formation at Marble Bar (Figure 5). Fluctuating pools filled by precipitation today support living microbial mat communities (Figure 5, left) and show evidence of microbial chemical staining when dehydrated (Figure 5, right). During the Archaean, organic compounds would accumulate in such pools and would be concentrated during cycles of dehydration by evaporation.

## 4. Fossil Stromatolites

**Figure 6:**
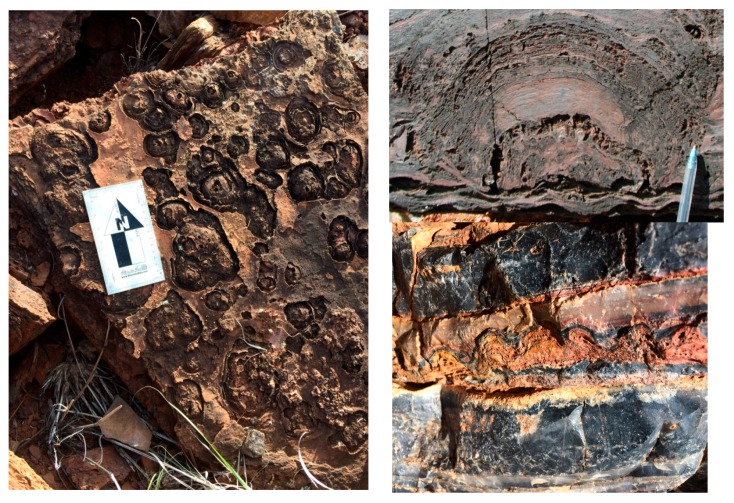
(**Left**) Author’s photo of overhead view of *ca.* 2.7 Ga domical stromatolites from the Tumbiana Formation, Fortescue Group. (**Right top**) Cross-section of a Tumbiana stromatolite (credit: Martin Van Kranendonk). (**Right bottom**) Author’s photo of cross-sectional view of centimeter-scale, domical stromatolites in black chert from the “Trendall locality” outcrop of the *ca.* 3.4 Ga Strelley Pool Formation on the Shaw River.

Well-preserved fossil stromatolites are abundant in the *ca.* 2.7 Ga Tumbiana Formation. These are “domical” (dome-shaped) stromatolites (Figure 6), which bear a clear resemblance to cross-sections of the stromatolites at Hamelin Pool (Figure 2, center). The field trip also toured the oldest rocks of the Pilbara region, the Apex chert, Strelley Pool and Dresser formations. Figure 6 (right bottom) shows a cross-sectional view through *ca.* 3.4 Ga stromatolites between beds of black chert at the “Trendall locality” on the Shaw River. Morphological similarity with modern day stromatolites at Shark Bay suggests that these structures are indicative of biogenicity [17,18,19]. Biogenicity is also supported by the fact that black chert from these localities has preserved rare microfossils [20]. Whereas the stromatolites of the Strelley Pool Formation formed in a shallow marine environment [21] with at least a local influence of hydrothermal activity [22], the *ca.* 3.48 Ga Dresser Formation, which contains the oldest convincing evidence for life on Earth [23,24], was deposited within an active volcanic caldera. The presence of geyserite provides clear evidence of extensive, fresh water hydrothermal systems on an exposed land surface, including geyser activity [25]. Furthermore, concentrated boron occurs in tourmaline-rich crusts, together with phosphate in hydrothermal veins, and clays from acid-sulfate hydrothermal alteration. These mineral components are considered to be cofactors of prebiotic chemical reactions related to life’s origin [14].

## 5. A novel Model for a Terrestrial Origin of Life

*“It is often said that all the conditions for the first production of a living organism are present, which could ever have been present. But if (and oh what a big if) we could conceive in some warm little pond with all sorts of ammonia and phosphoric salts, light, heat, electricity etcetera present, that a protein compound was chemically formed, ready to undergo still more complex changes [...]”* ~Charles Darwin, in an 1871 letter to Joseph Hooker [26].

The above speculation by Charles Darwin on where and how life might have begun is prescient and deeply insightful: a warm little pond in which a useful polymer forms and evolves to ever increasing complexity of structure and function. By expanding Darwin’s insight with today’s knowledge of chemistry and biochemistry, it is possible to propose a novel scenario for the origin of cellular life in a small, hot, cycling pool [27]: *A source of energy acts on a mixture of simple organic compounds to produce a large number of random polymers. By chance, a few rare polymers happen to form protocells defined as functional systems which are encapsulated within membranous compartments that are subject to combinatorial selection. The encapsulated polymer systems continuously cycle within a kinetic trap in which the rate of synthesis exceeds the rate of hydrolysis [28,29]*. *Over a large number of such cycles, populations of protocells emerge that are capable of growth and replication. Combinatorial selection of protocells that are better able to survive environmental stresses and more efficient in capturing energy and nutrients, drives the evolution of populations toward the first primitive forms of life.*

It is difficult to imagine how the above scenario could be applied to an origin of life at marine settings such as hydrothermal vents. First, the continuous presence of water puts thermodynamic limits on the condensation reactions that are required for polymerization of monomers. Furthermore, high concentrations of salt and divalent cations at vents or tide pools impose osmotic stresses on membranous compartments. Life today has evolved complex homeostatic mechanisms to regulate cell volume by actively pumping osmotically active ions and thereby maintain transmembrane sodium and potassium gradients as noted by Mulkidjanian *et al.* [10].

These concerns led to consideration of an alternative scenario that life began in fresh water hydrothermal fields subject to cycles of dehydration and hydration as outlined by Deamer and Georgiou [30]. To summarize briefly, given a process that could synthesize functional polymers, the polymers become enclosed within compartments, referred to as protocells, in order to prevent dispersion into a bulk aqueous phase. The polymers would resemble RNA and peptides, as well as possible complexes of RNA and peptides. The boundary membranes of protocellular compartments must be selectively permeable to permit access to nutrients from the surrounding environment. A single protocell containing random polymers cannot evolve biologically relevant functions in isolation. Instead, a combinatorial process is necessary to generate large populations of protocells, test the functional fitness of encapsulated systems of polymers, and permit these polymers to be expressed in later cycles of the population. The transition to life is a continuum, along which the synthesis of functional polymers moves beyond physical self-assembly and comes under the control of an autonomous set of molecular processes driven by instructions, itself subject to replication, mutation and propagation through generations of protocells.

## 6. Linking Chemical Reactions to the Physical Properties of Hydrothermal Pools

**Figure 7:**
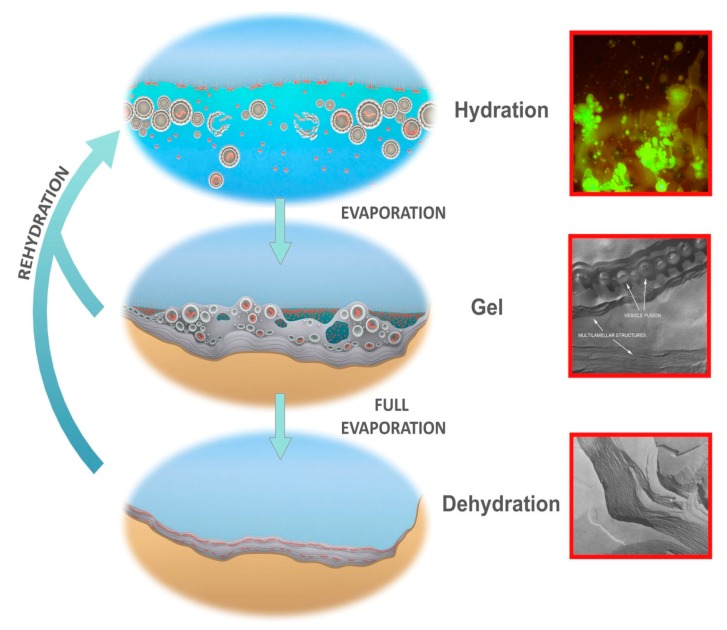
Protocells cycling between three coupled phases. Hydrated phase (**Top**), inset: protocells containing DNA growing out of a dried mixture of DNA and phospholipid, hydrated by water, stained with acridine orange. Gel phase (**Center**), inset: hydrogel of lipid with approximately 50% water by weight showing lipid vesicles fusing into lamellae. Dehydrated phase (**Bottom**), inset: freeze fracture image of anhydrous lipid lamellae of phosphatidylcholine. Micrographs credit: David Deamer.

Hydrothermal pools may contain mixtures of organic solutes, some of which are potential monomers, and others capable of forming membranous vesicles. During the evaporation that drives the dehydration phase of a cycle, layers of lipid membranes form films on mineral surfaces at the pool edge as the water level fluctuates. Amphiphilic molecules assemble into multilamellar structures in which monomers are captured between the lipid bilayers [31]. Figure 7, bottom inset shows the multilamellar character of one such amphiphile, in this case anhydrous phosphatidylcholine. The concentrated monomers (shown in red) are organized within a two-dimensional reaction space, and reduced water activity drives condensation reactions in which water becomes a leaving group, resulting in synthesis of ester bonds and peptide bonds, the primary links of biopolymers today. Assuming that amino acids and nucleotides are among the potential reactants [32], the synthesis of peptide bonds and phosphodiesters will occur and this has recently been demonstrated in laboratory simulations [33,34,35,36,37]. By chance, some of the polymers will have functions that are relevant to selection as components of protocells.

Short term dehydration and rehydration of the pool through periodic fluctuations in hydrothermal water supply, rainfall events, and dew from day-night cycles or through changing humidity cause the outer layers of the dried lamellae to disperse large numbers of protocells into the bulk solution. Some of these protocells will contain random sets of polymers (Figure 7, top-inset shows DNA encapsulation). The protocells are then subject to selective processing in which the aqueous environment disrupts some protocells while encapsulated functional polymers stabilize their membranous compartments and thereby increase their robustness [38]. Stabilized protocells would persist and return to deposit their surviving cargos of polymers back into the lamellae as protocells dry down and undergo fusion. In this manner the polymers are “coupled” between phases and cycled within a kinetic trap, permitting generations of polymers to be re-synthesized, tested and, as Darwin intuited, become increasingly complex.

The kinetic trap provides scaffolding for the stepwise emergence of encapsulated systems of functional polymers described earlier in the *coupled phases* scenario proposed by Damer and Deamer [27]. Functions emerge as selective pressures present hurdles for populations of protocells undergoing chemical evolution. The hurdles can be surmounted by the stepwise accumulation of functions such as membrane stabilization; osmotic equilibration and transport by transmembrane pores; primitive metabolism utilizing nutrients and energy sources available in the environment; feedback controls; and catalytic cycles enabling replication of polymers. For Darwinian selection to begin to operate, simple self-assembly must be supplemented and ultimately replaced by active processes encoded in informational polymers. The RNA-World hypothesis [39,40] suggests that transfer of proto-genomic sequence information could be first expressed by simple oligonucleotide templating [41] then later through the combinatorial selection of a pool of functional ribozymes [42] some of which are capable of RNA-catalyzed template-driven synthesis of other RNA sequences by active ribozymes [43,44]. These laboratory tests of RNA World concepts require cycling, interaction and combinatorial selection of large numbers of polymers encapsulated in glassware. A natural kinetic trap of the kind described above may have supported some version of these powerful laboratory demonstrations of molecular evolution and brought an RNA World, or perhaps more likely, an RNA-Protein World, into existence.

## 7. A Gel Phase as Candidate Progenote

It is a common observation that terrestrial microbial life in the form of mats is metabolically most active in a moist, gradually dehydrating state following a short term hydration event such as rain fall [1]. This property suggests a link with an earlier form which can only occur in fluctuating pools: a hydrogel that emerges naturally when sufficiently large populations of stable protocells crowd together next to mineral surfaces as the pool water level drops. Figure 7, center-inset illustrates lipid vesicles (~50% water by weight), which aggregate and form such a “gel phase” during dehydration. In a hydrothermal pool such gel structures would contain concentrated organic solutes and mineral grains along with systems of randomly synthesized functional and inert polymers. A self-assembled gel aggregate can act as a scaffold for the emergence of key biological functions as suggested by Tevors and Pollack [45], and possesses properties suggesting evolutionary continuity [46] with later microbial communities. A gel aggregate also represents the first example of niche construction described by Odling-Smee [47] in which a micro-environment is modified for the benefit of its members in a primordial example of endosymbiosis [48,49,50].

**Figure 8:**
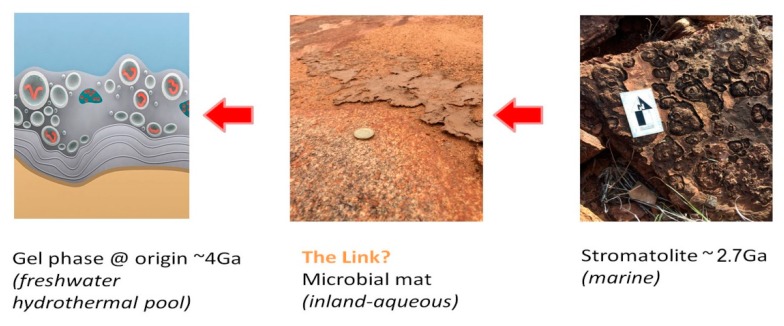
Desert microbial mats (**Center**); suggestive of an intermediate form between marine stromatolites (**Right**); and an ancestral form of all microbial communities (**Left**).

A gradient of selective pressures would be required to give rise to adaptations that enable the transition from a non-living gel phase to a primitive microbial population. Examples of such selective pressures are exposure to environmental gradients such as pH, temperature, nutrient and salt concentrations. Stromatolite fossils observed today were presumably produced by complex microbial communities with a number of finely tuned adaptations (Figure 8, right). These include mat formation using an excreted polymeric substance, specialization of community members carrying out photosynthesis, chemosynthesis and other metabolic functions, cementation of mineral sediments, and tolerance for fluctuating hydration and salinity [51,52]. It follows that the microbial populations forming stromatolites able to tolerate Archaean marine shorelines had advanced far along the evolutionary gradient.

A predecessor would therefore possess a subset of these adaptations. The microbial mats observed at Gallery Hill (Figure 8, center) are suggestive of intermediate form between an earlier ancestor and a marine stromatolite. However, it should be noted that microbial communities as represented by living examples today as well as in the earliest fossil record arose long after LUCA, the last universal common ancestor. The path from an early form of life to autonomous cellular structures defined as LUCA is not expected to be tree-like, but will more likely resemble the shrub metaphor of Doolittle [53].

A gel phase (Figure 8, left) can retain moisture and concentrate nutrients, providing for the emergence, testing and sharing of polymer functions such as membrane stabilization and pores required for solute transport in and out of protocells. With nutrient solute transport come opportunities to support primitive metabolic processes within protocells. A gel is also an ideal venue for protocell competition [54,55]. Furthermore, a gel would tend to retain the components of disrupted protocells, thereby selecting for surviving protocells that can absorb these in a primitive form of heterotrophy. A gel would also help to maintain a collective transmembrane solute balance, thereby protecting all its member protocells against osmotic stress. Lastly, a gel would be subject to group selection in which a metabolic product or functional innovation from one protocell would diffuse throughout the gel, benefiting the entire population suggesting an early pattern resembling horizontal gene transfer, central to microbial evolution today.

I propose that the protocell gel phase is a candidate *progenote* as described by Woese and Fox [56,57], in which its member protocells are engaged in the *collective process of evolving the relationship between genotype and phenotype*. As individual protocells gradually accumulate and internalize proto-genomic functions, they will develop more autonomous phenotypic expression. A new evolutionary phase of the progenote will arise when member protocells can enzymatically replicate encapsulated genomic and functional polymers and pass them on to daughter cells without depending on dehydration synthesis. The resulting speciation and specialization of increasingly independent protocells will lead to communities becoming robust to steeper gradients of environmental pressures. Some individual protocells will eventually become advanced enough to survive and reproduce independent of their source community, passing an important milestone on the continuum to life. Wider distribution and direct competition between free-living cells will drive this early life along the long winding road to LUCA.

## 8. The Adaptive Radiation of the Progenote

**Figure 9:**
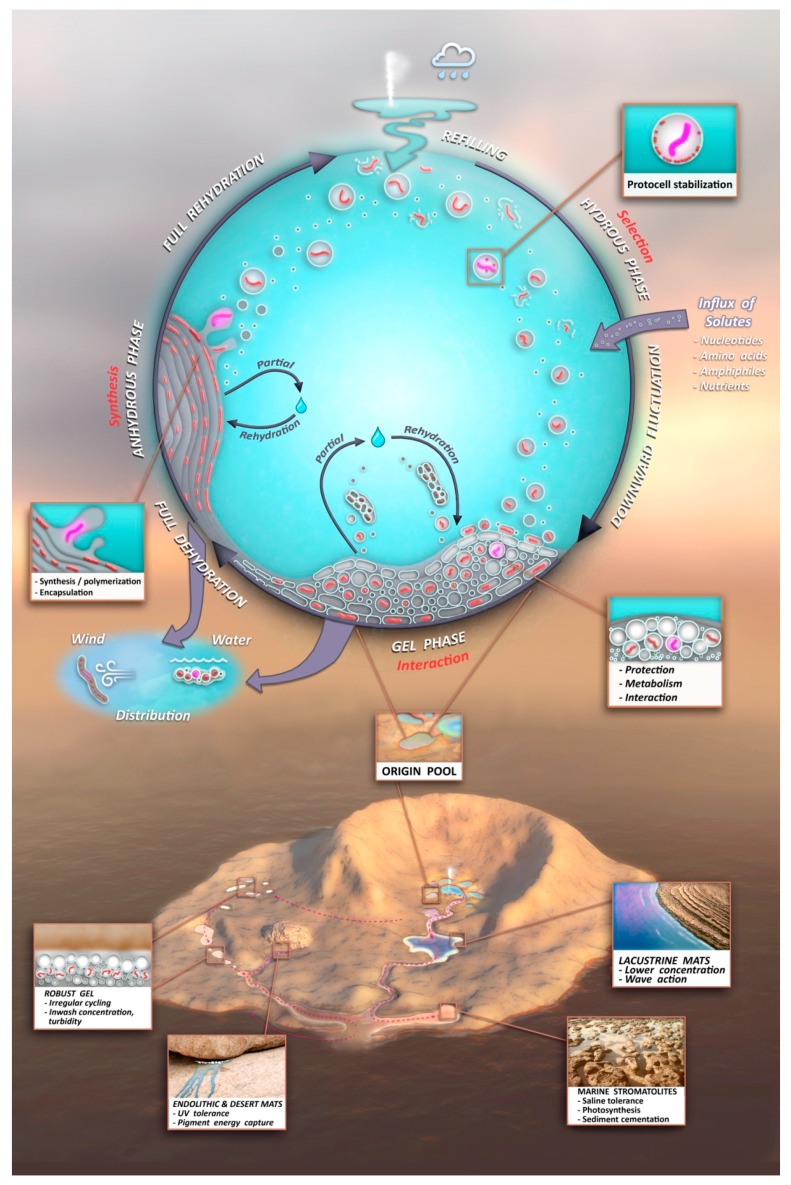
Overview of the unified chemical (**Upper**) and geological (**Lower**) contexts and their interface through protocell gel progenote distribution (**Center**) comprises the model of the *terrestrial origin of life hypothesis*.

A protocell gel progenote possesses one more property that supports its central role in the origin of life: mobility. As soon as the components cycling in a hydrothermal “origin pool” (Figure 9, upper) reach a steady state incorporating a kinetic trap, they will give rise to a robust, growing and adaptable progenote. This has the potential to be distributed (Figure 9, center left) as a raft, transported by gravity downstream in its hydrated phase, or as a film detached by wind from mineral surfaces and dispersed in the dry phase. Distribution is essential because the initial sustaining ‘Goldilocks’ conditions at the origin pool may not persist. When they arrive at new aqueous venues dispersed throughout the geological context of an Archaean volcanic island (Figure 9, lower), progenote aggregates will continue to undergo adaptive radiation by responding to environmental pressures and accumulating innovations through individual and group selection. Innovations will be continuously mixed and shared by combining functions from different gel aggregates. The lower figure insets illustrate some informed guesses as to the effect of various geological venues in the downhill journey of the progenote as it evolves toward the living microbial communities which ultimately build the stromatolites so abundant in the earliest fossil record. This unified view of chemical and geological contexts completes a high level treatment of the *terrestrial origin of life hypothesis*, offered as a model to the astrobiology community.

## 9. Summary of Novel Features and Testable Predictions of the Model

The novel features of the model are summarized below, each carrying predictions that can be subject to testing by experimentalists:

- A kinetic trap cycling polymers through coupled phases of hydration and dehydration will produce large numbers of random molecular systems synthesized between dehydrated lipid lamellae (Figure 9, upper left), which become encapsulated within large numbers of lipid compartments (protocells).
- Combinatorial selection of encapsulated functional polymers will be observed within this kinetic trap. The initial selection criterion will be the ability of encapsulated polymers to enhance the stability of their membranous compartments, which will be tested in the hydrous phase (Figure 9, upper right).
- During dehydration, stable protocells will crowd together with concentrated solutes forming a third, gel phase (Figure 9, center) which provides a protective environment. Interaction of protocells within the gel will enable early forms of metabolism, competition, and sharing of functions and products across the gel.
- These three phases will provide continuous resources and subject molecular systems to a variety of selective pressures, driving the system to surmount hurdles to achieve stepwise molecular evolution of new functions.
- Pure self assembly and random synthesis will enable the initial stages of the model, but eventually, active functions expressed through sets of robust, heritable proto-genomic instructions will enable Darwinian evolution to take hold.
- Degradation rates of synthesized polymers will set upper bounds on the permitted dwell time within each phase suggesting the necessity of reliable, repetitive fluid refilling typical of hydrothermal fields. Expressing a pattern emerging later as the life cycle, degraded polymers and other inert byproducts must be periodically reused or expelled and functional systems continually re-synthesized through proto-genomic blueprints drawing from solute sources (Figure 9, upper right).
- A candidate protocell gel progenote capable of growth and evolutionary adaptation will emerge and become robust to distribution to a variety of watery venues in a plausible Archaean volcanic landscape. Extensive laboratory growth, testing, and analysis of such progenotes will provide insight to possible pathways to the first microbial communities and viable free-living cells.

## 10. Conclusions

A field trip in Western Australia to venues ranging from living marine stromatolites to inland Archaean stromatolite fossil localities tested the *coupled phases* scenario for an origin of life. A novel *terrestrial origin of life hypothesis* was developed through the unification of chemical and geological settings. The model is centered upon the insight into a protocell gel progenote, a candidate common ancestor of microbial communities. It seems inescapable that very soon after the first robust protocells emerged, they became communal. Primitive protocells floating free in the Archaean environment would be unlikely to survive, while selection would favor the protective, nutritional and collaborative advantages of a protocell gel progenote. This progenote is a model for an amphibious, communal aggregate of protocells that preceded LUCA, a much more highly evolved population of cells able to independently support their own metabolism and reproduction. The progenote described here represents a common pool, or roots from which the earliest inhabitants of the tree of life emerged.

## Resources

A website has been established to support collaboration, testable hypotheses and research results on the terrestrial origin of life hypothesis at: http://origins.biota.org.
